# Rationale for Combining Bispecific T Cell Activating Antibodies With Checkpoint Blockade for Cancer Therapy

**DOI:** 10.3389/fonc.2018.00285

**Published:** 2018-07-25

**Authors:** Sebastian Kobold, Stanislav Pantelyushin, Felicitas Rataj, Johannes vom Berg

**Affiliations:** ^1^Center of Integrated Protein Science Munich and Division of Clinical Pharmacology, Department of Medicine IV, Klinikum der Universität München, Munich, Germany; ^2^Institute of Laboratory Animal Science, University of Zurich, Zurich, Switzerland

**Keywords:** anti-CTLA4, anti-PD1, bispecific T cell activating antibodies, immune checkpoint blockade, combination therapy, cancer immunotherapy, anergy

## Abstract

T cells have been established as core effectors for cancer therapy; this has moved the focus of therapeutic endeavors to effectively enhance or restore T cell tumoricidal activity rather than directly target cancer cells. Both antibodies targeting the checkpoint inhibitory molecules programmed death receptor 1 (PD1), PD-ligand 1 (PD-L1) and cytotoxic lymphocyte activated antigen 4 (CTLA4), as well as bispecific antibodies targeting CD3 and CD19 are now part of the standard of care. In particular, antibodies to checkpoint molecules have gained broad approval in a number of solid tumor indications, such as melanoma or non-small cell lung cancer based on their unparalleled efficacy. In contrast, the efficacy of bispecific antibody-derivatives is much more limited and evidence is emerging that their activity is regulated through diverse checkpoint molecules. In either case, both types of compounds have their limitations and most patients will not benefit from them in the long run. A major aspect under investigation is the lack of baseline antigen-specific T cells in certain patient groups, which is thought to render responses to checkpoint inhibition less likely. On the other hand, bispecific antibodies are also restricted by induced T cell anergy. Based on these considerations, combination of bispecific antibody mediated on-target T cell activation and reversal of anergy bears high promise. Here, we will review current evidence for such combinatorial approaches, as well as ongoing clinical investigations in this area. We will also discuss potential evidence-driven future avenues for testing.

## Introduction

Since its inception in the 1940s, drug-based cancer therapy has been centered on targeting the cancer cell through different strategies aiming at reducing their growth (1). With the development of recombinant techniques and the hybridoma technology (2) for the generation of monoclonal antibodies, therapies utilizing the immune system have entered the clinical realm from the 1990s (3, 4). However, the main target of antibodies remained the cancer cell or cancer cell associated processes such as growth factors (5). The clinical value of these advances is unchallenged and has enhanced patients' prognosis in a number of indications.

More recently, a paradigm change has occurred in clinical oncology, establishing the immune system in general and T cells in particular as therapeutic effectors. Antibodies targeting and activating T cells have been approved for the treatment of advanced cancer types such as metastatic melanoma, advanced non-small cell lung cancer or renal cell carcinoma (6, 7). This advance has been made possible through the recognition that cancer cells suppress the immune system, and especially adaptive anti-tumoral immunity to progress to overt clinical disease (8). In this process, suppression of T cell function and recognition of cancer antigens through induction of T cell anergy or dysfunction has been identified as an essential step. Based on these seminal discoveries, antibodies neutralizing the anergy mediating or T cell suppressing molecules PD1, PD-L1 or CTLA4 have entered clinical practice (6, 7). These antibodies have led to unparalleled response rates and even cures in previously untreatable medical conditions, including advanced metastatic melanoma (9). As especially the PD1-PD-L1 axis appears to be a central process across cancer entities, it is not surprising that the antibodies nivolumab, pembrolizumab (both anti-PD1) or atezolizumab, avelumab and durvalumab (anti-PD-L1) are approved for a growing number of indications based on efficacy (10). Due to their mode of action, these drugs are now being used in combination trials both with conventional treatments such as chemo- or radiotherapy, as well as other immunotherapeutic strategies in over 1,000 open clinical trials (10). A major limitation of checkpoint blockade is the specificity of the approach, as any T cell encountering its antigen outside of the tumor tissue may be unleashed. While this is highly desirable in terms of breadth of the anti-tumoral immune response, a significant issue are autoimmune side effects which can be life threatening (9).

Another, potentially more selective, approach to redirect T cells against cancer cells are bispecific antibodies (BiAb) (11). BiAb can bind two antigens simultaneously and can act as a bridging agent for two different cell types. One of the most widely used concepts are T cell-activating bispecific antibodies (TABs), which would activate T cells in the vicinity of cancer cells targeted through simultaneous binding of a tumor associated antigen (TAA) (12). For the purpose of this review we will use the short form TAB for any bispecific molecule activating T cells, irrespective of the format or the target molecule. A TAB targeting CD19 and CD3 (blinatumomab) effectively redirects T cells against acute lymphocytic leukemia (ALL) cells and induced remission in refractory patients (13). This has led to its approval for the treatment of certain ALL types. Many other TABs are under investigation for several indications (11). However, even in the context of ALL, their activity appears to be limited and additional strategies are required to enable their use in a broader clinical setting (14). In the present review, we will give an overview of current developments in the TAB field, identify resistance and escape mechanisms that need to be overcome to enable TAB activity and summarize data on most advanced combination strategies utilizing TAB together with checkpoint blockade.

## T cell engaging bispecific antibodies for cancer therapy

**Figure 1:**
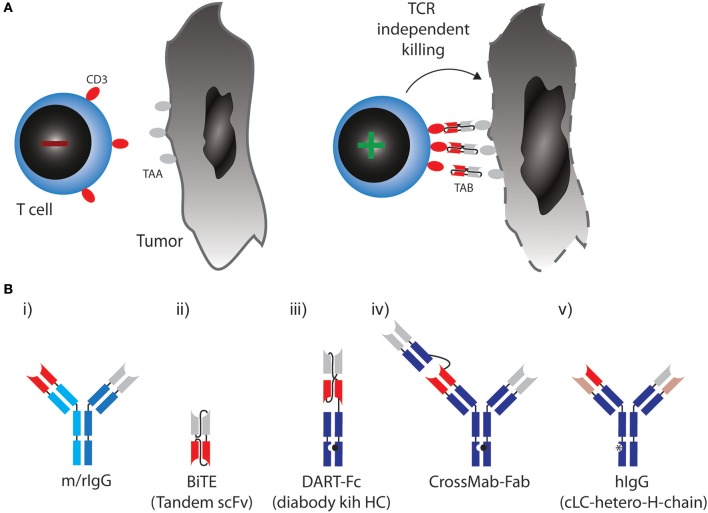
**(A)** Therapeutic concept utilized by TABs: Binding of tumor associated antigen (TAA) on cancer cell leads to crosslinking of CD3 on T cells, activation irrespective of TCR specificity and tumor cell lysis. **(B)** Molecular formats of TABs which are approved or currently tested in combination with checkpoint blockade. (i) Bispecific rat/mouse chimeric, quadroma derived antibodies with 1:1 valency, e.g., Catumaxomab. (ii) BiTE: two single chain variable fragments (scFv) connected via flexible linkers as a continous polypeptide with 1:1 valency, e.g., Blinatumomab. (iii) DART-Fc: two VL chains that each have their corresponding VH chains interchanged and are expressed as two separate chains. One of the chains has a knob-Fc domain, a third chain entails the whole-Fc domain, 1:1 valency, such as MGD007. (iv) CrossMAB-Fab: heterodimeric constant light chain assembly combined with knobs-into-holes mutations for heterodimeric heavy chain pairing with 1:2 valency, e.g., CEA-TCB. (v) Bispecific fully human IgG format (hIgG) with a common light chain and heterodimeric heavy chains with 1:1 valency, e.g., REGN1979.

In the 1960s, the first reports on bispecific antibody derivatives were on antigen-binding fragments (Fabs) from two different polyclonal sera re-associated into bispecific F(ab')_2_ molecules (15). The development of the hybridoma technology in 1975 allowed researchers to produce large amounts of monoclonal and later bispecific antibodies (2, 16, 17). The advent of engineered bispecific antibody formats set the stage for applications beyond simple antigen neutralizing, antagonistic or agonistic binding. Over three decades of research have come up with more than 100 molecular formats (18). At least a quarter of those formats have also been used to design TABs (19). For space reasons only approved formats and designs currently being clinically tested in combination with checkpoint blockade are described in this review (Figure 1). A comprehensive overview of all other formats can be found in Wu and Cheung (19).

In most formats in clinical development, a monovalent binder for CD3 is combined with a monovalent TAA binding site (a 1:1 valency). Apart from valency, the TAB's affinity for CD3 is designed so that it does not trigger T-cell receptor signaling through CD3, unless it is presented to the T cell in a multivalent fashion bound to a TAA on a target cell [Figure 1A, (12)]. In any case, T cells are redirected to a TAA regardless of their initial specificity, can exert direct cytoxicity and induce cytokine responses triggering further bystander activation.

The first clinically approved TAB was Catumaxomab (Removab, a bispecific IgG antibody) in 2009, targeting CD3 and epithelial cell adhesion molecule (EPCAM) for the treatment of malignant ascites. Catumaxomab is a trifunctional antibody, consisting of mouse IgG2a (EPCAM) and rat IgG2b (CD3) (Figure 1B) (i) and is produced using quadroma technology (20). While Catumaxomab was voluntarily withdrawn from the market in 2013, two more TABs based on the same format, targeting CD20 or Her2 have been tested in early phase clinical trials (21, 22).

A very small format to generate 1:1 valency are bispecific T cell engagers (BiTEs). BiTEs are also known as tandem single chain variable fragments (scFvs) and are composed of two scFvs, each with a unique antigen specificity (Figure 1B) (ii). The entire BiTE molecule consists of one continuous polypeptide of ~55 kDa, compared to 150 kDa for a conventional IgG antibody (23). As with full IgG format TABs, one scFv in BiTEs targets the CD3 and the other scFv targets a TAA.

Blinatumomab (Blincyto, Amgen) became the first and so far only clinically approved BiTE for the treatment of ALL. It engages T cells through CD3 binding, while the other scFv is specific for CD19, expressed by B cells, including B-lineage leukemias and lymphomas (24, 25). In a phase II trial, Blinatumomab achieved complete remissions in 69% of patients with relapsed or refractory ALL (26).

Even smaller than BiTEs, dual-affinity retargeting (DART) proteins have a diabody format where one VL chain is followed by the VH chain of the second binder and the two polypeptide chains align in a head-to-tail fashion. DARTs also face the problem of low *in vivo* half-life, which can be partially solved by fusion of an Fc domain (DART-Fc, Figure 1B) (iii) (27).

While the above formats all use symmetric design to create 1:1 valencies for CD3 and TAA targeting, evidence suggests that 1:2 design with two binding sites for the TAA and one for CD3 might be beneficial to generate strong binding to tumor cells while avoiding CD3 activation in the absence of TAA. A versatile format termed CrossMab technology enables the heterodimeric constant light chain assembly and together with the knobs-into-holes method to generate heterodimeric heavy chain antibodies, which allows not only the generation of bispecific antibodies in full IgG format, but also 1:2 valencies (Figure 1B) (iv) (28). This method was used to develop a TAB, which binds CD3 and carcino-embryonic antigen (CEA), with a 1:2 valency [Figure 1B, (29)]. Most TABs use fully human BiAb formats with near-native antibody architecture (Figure 1B) (v). Currently, 10 different IgG format TABs are being clinically tested (19).

## Limitations and resistance mechanisms to TABs

To date catumaxomab and blinatumomab are the only TABs that have achieved regulatory approval. Due to the premature withdrawal of the former, our knowledge of the TAB limitations comes primarily from traditional monoclonal antibodies and blinatumomab (13).

Moving from hematological malignancies onto solid tumors, a major limitation of all antibodies is their (in-)ability to reach their target. While sites such as lymph nodes and the bone marrow have excellent accessibility, it is lower for other tissues such as synovial joints and the kidney. For the central nervous system (CNS), antibody drug exposure can be <1% relative to systemic circulation. Poorly organized vasculature also limits blood flow rates and contributes to inefficient drug delivery in solid tumors (11, 30). To date, no clinical successes using TABs in solid tumors have been reported. Dose-limiting toxicity and low half-life can be prohibitive for the use of BiTEs in such tumors. Sufficient dosing to reach poorly perfused tumors without causing serious adverse events (AEs) is challenging. Another problem with non-lymphoid tumors is that TAAs are often not exclusively expressed on transformed cells, raising the issue of on-target but off-tumor toxicities which can be dose and efficacy limiting.

A crucial issue with the polyclonal activation of T cells by TABs, independent of TAA binding, is a potentially fatal cytokine release syndrome (CRS) similar to the adverse events observed with a CD28 superagonist antibody (14, 31). The CRS goes hand in hand with disease load in patients and correlates with dosage, in turn limiting application either to lower dosage or to patients with lower tumor burden.

TAB therapies also run into the issue of tumor mutations and subsequent treatment escape. For blinatumomab, about 15% of patients experience CD19-negative relapses of ALL due to a disrupted CD19 membrane export (32). In such patients, blinatumomab selects for CD19-negative ALL cells and prevents further BiTE activity. A notion that might counteract this limitation is epitope spreading occurring under active immunotherapy. Evidence for epitope spreading comes from preclinical studies with catumaxomab and a BiTE targeting an intracellular oncoprotein (33, 34). However, the setting where blinatumomab is applied might not be beneficial for epitope spreading as these patients frequently have pancytopenia either as consequence of disease or treatment and might not be able to mount an effective T cell response.

**Figure 2:**
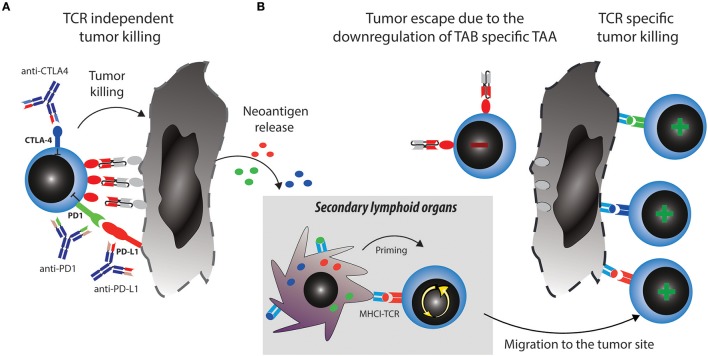
Strategies to overcome tumor escape mechanisms through combining TABs with checkpoint blockade. **(A)** Activated T cells upregulate checkpoint molecules such as PD1 and CTLA4, which can lead to their suppression and anergy, allowing tumors to escape. Combination therapy of TABs with checkpoint blockers unleashes suppressed T cells and restores tumor cell killing via TAB. This in turn releases new tumor antigens. **(B)** Tumor antigens are taken up by antigen presenting cells (APCs) and prime new T cell clones, this broadens the antigen specific T cell response and leads to tumor eradication through antigen spreading even if the tumor downregulates TAB specific TAA.

Two major reported ALL escape mechanisms during treatment with blinatumomab included increased frequencies of regulatory T cells (T_regs_) (35) and increased levels of PD-L1 expression on B-precursor ALL cells (36). T_regs_ suppress effector T cell activation through CTLA4 and other mechanisms [reviewed in (37)]. However, even when T cells get fully activated, upregulation of PD1 will lead to inhibitory signaling after binding to PD-L1 expressed by the tumor cells. These mechanisms induce effector T cell suppression and exhaustion or dysfunction, which can be therapeutically countered with checkpoint blockade (Figure 2A).

## Combination of TABs with checkpoint blockade

The blockade of the PD1—PD-L1 axis restores blinatumomab activity *in vitro* (38). Comparable data has been described with the anti-CD3 × anti-CD33 BiTE AMG330 (39). AMG330 upregulated PD1 on T cells and PD-L1 on AML blasts *in vitro*. Lytic potential, T cell activation and proliferation are strongly enhanced upon blockade of the PD1-PD-L1 axis (39, 40). Addition of costimulatory agonistic anti-CD28 antibodies to AMG330/T cell/blast coculture boosted blast lysis (40). In line with these results, using a novel anti-CD3 × anti-CD307 bispecific antibody, suppression of T cell mediated killing was observed on myeloma cells through PD-L1 and selective antibody-mediated blockade restored T cell activity (41). Finally, the use of a bispecific single chain antibody converting negative PD-L1 signaling into positive costimulation through CD28 on T cells has been shown to improve the activity of blinatumomab *in vitro* (42). These cancer entities and molecule spanning synergies underpin the relevance of the combination of PD1-PD-L1 disruption and bispecific T cell activating antibodies in hematologic malignancies. They have prompted the design and the initiation of ongoing clinical studies combining blinatumomab with checkpoint inhibition (Table 1).

**Table 1:** Clinical trials testing TAB in combination with checkpoint blocking antibodies Tested compounds, molecular targets, format, indication and trial status are indicated.

**Compounds**	**Target**	**Format of TDB**	**Indication**	**Identifier**	**Status**
Blinatumomab and Pembrolizumab	CD19 and PD1	BiTE	Refractory or relapsed diffuse large B cell lymphoma	NCT03340766	Not yet recruiting
Blinatumomab, Nivolumab, Ipilimumab	CD19, PD1, CTLA4	BiTE	Refractory acute lymphoblastic leukemia	NCT02879695	Recruiting
Anti-CEA x anti-CD3 bispecific antibody and atezolizumab	CEA and PD-L1	CrossMAB-Fab	Advanced CEA+ solid tumors	NCT02650713	Recruiting
Anti-PD1 and anti-CD3 x anti-CD20 antibodies	CD20 and PD1	cLC-hetero-H-chain IgG	B lymphoid malignancies	NCT02651662	Recruiting
Anti-PD1 and anti-CD3 x anti-gpA33	gpA33 and PD1	DART-Fc	Refractory or metastastic colorectal cancer	NCT03531632	Recruiting

*A comprehensive overview of clinical trials using TAB alone or in combination with other than checkpoint inhibition is found elsewhere (19)*.

In other non-hematological entities, various TAB are under testing and development and it is not surprising that a potential synergistic activity with checkpoint inhibition is also being evaluated. As seen in hematological malignancies, both PD1 on T cells and PD-L1 on tumor cells are upregulated upon treatment with TABs (29, 43). However, the value of blockade of PD1—PD-L1 interaction is more controversial in such indications and might depend on the molecule used, as well as the tumor site. Activity of anti-CD3 × anti-CEACAM5 × anti-Trop2 antibody was enhanced when combined with PD1-blockade *in vivo* (44). PD1-PD-L1 inhibition also enhanced lysis mediated by anti-CD3 × anti-CEA bispecific T cell engager but was unable to restore T cell activity completely upon induced T cell exhaustion (45). These later results point toward additional mechanisms impairing T cell activity under these conditions. Along these lines, exhausted T cells have also been described to have reached a state where such combination alone can no longer convert them into an active T cell (46). In such a situation, a TAB may even have detrimental activity in conjunction with other treatment modalites and worsen activation induced cell death in combination with radiation. Combination of an anti-CD3 × anti-CD133 bispecific antibody with radiation accelerated tumor growth due to cell death, which could only be partially prevented by PD1 blockade (47). Another important aspect seems to be the exact antibody format or targeting moiety used, as some TABs targeting HER2 on breast cancer cells are found to be insensitive to PD1-PD-L1 mediated T cell suppression toward their activity, while blockade of the axis might enhance the lytic potential of another anti-HER2 TAB (48, 49).

While so far a major focus of research has been on the PD1-PD-L1 axis based on the mode of action which is predicted to be at the tumor site, the use of the other approved checkpoint axis blocker against CTLA4 (ipilimumab) has also been investigated. CTLA4 blockade ameliorates the activity of TABs, although to a more modest extent than seen with PD1 or PD-L1 blockade (33). Some studies, also indicate that blockade of both axes is required to achieve superior tumor cell killing (46).

Preliminary clinical results have been reported for the combination of atezolizumab (anti-PD-L1) with anti-CD3 × anti-CEA TCB and for an anti-PD1 antibody together with anti-CD3 × anti-CD20 bispecific antibodies in colorectal carcinoma and B cell lymphoma respectively (50, 51). Both studies have disclosed signs of activity and responses, indicating that the combination will also be valuable clinically. Longer follow up and full disclosure of the results will be required to assess the clinical value of the strategy.

A major mechanistical aspect from such a combination strategy, which can prevent resistance and escape is the induction of epitope spreading. Tumor reduction by TABs will make tumor antigens accessible to the immune system and enable induction of specific T cells which can be unleashed or further boosted by checkpoint blockade (Figure 2B). Vice versa, a similar mechanism is envisionable when tumor reduction is propelled by checkpoint blockade and immune response is boosted by the TAB. Preclinical examples of epitope spreading have been reported for BiTE and checkpoint blockade (34, 52, 53), paving the way for the concept that epitope spreading might be most prominent when both modalities are combined (Figure 2). On the other hand, checkpoint blockade targeting for example CTLA4 has been reported to be most effective when preexisting immunity against TAA was detected at baseline (54). A notion that is countered by others, as similarly, *de novo* induction of anti-tumor responses have been described to be the best predictor of clinical activity (55). Along the same lines, both *de novo* and preexisting immunity is associated with treatment response to PD1-blockade (56). Existing evidence thus points toward epitope spreading as an important determinator of response to immune checkpoint blockade. Clinically, mutational load is a predictive marker for response to checkpoint blockade. Similar thoughts would thus conceptualize the notion that more mutations provide more targets for T cells and thereby a better epitope spreading (57). As both checkpoint blockade and TAB are associated with the occurrence of epitope spreading to varying degrees, we argue that combinations will enhance the likelihood of this important mode of activation to happen. It would also come with the advantage of prolonged activity over time even when the drugs are discontinued and to potentially reduce the occurrence of antigen-loss variants. Evidence for the benefit of this strategy and for its mode of action will come from future and ongoing clinical trials.

## Risks associated with the combination of TAB and checkpoint blockade

Most of our knowledge on the side effects to be expected by the clinical use of TABs comes from the use of blinatumomab. The side effects are considerable with over 80% of patients experiencing side effects of grade three or above (side effect requiring hospitalization and/or life threatening) (58). Apart from neutropenia, infections and other common side effects in hemato-oncology, blinatumomab is also associated with severe CRS and neurological symptoms of unknown ethiology. In specialized centers these are in general manageable and eventually completely resolve in most patients. In contrast, severe side effects of grade three or above are typically less frequent when targeting the CTLA4 or PD1 pathway with checkpoint blockade (~28 and 21%, respectively) (59). The safety profile is, however, very distinct with mainly autoimmune related side effects including colitis and polyendocrinopathies. These autoimmune phenomena can be managed if recognized and treated adequately but might also lead to the need of life long hormone substitutions in a number of cases when endocrine organs are affected. Limited data exists for the potential safety profile of the combination of TAB with checkpoint blockade in clinical trials. The combination of the CEA TCB with anti-PD-L1 blockade suggest that toxicities of either agent do not multiply and the most frequent adverse events so far were infusion related reactions and diarrhea (60). However, this data need to be interpreted with caution, as so far only published as a conference abstract and experience with combined checkpoint blockade suggests that toxicities from immune active agents might at least add up to each other for a more detrimental safety profile. A longer follow up and results from ongoing prospective trials are expected to answer these questions.

## Conclusion and outlook

T cell activating bispecific antibodies have considerable activity in refractory B-ALL but only a fraction of the treated patients will benefit from it in the long run. A major mechanism limiting the activity of bispecific antibodies in ALL and other indications appears to be T cell anergy and exhaustion driven by, among others, the PD1-PD-L1 axis. Preclinical evidence suggests that bispecific antibody activity under these settings can be restored or even enabled when combined with antibodies to checkpoint molecules. Under this combination, induction of epitope spreading may be an important mode of action of a combinatorial treatment. However, current evidence also indicates that not all bispecific antibodies, nor all indications will benefit from such combinations forcing the need for more detailed research. Although outside of the scope of the present review, it is important to note that bispecific T cell engaging antibodies might also act in synergy with other immunotherapeutic modalities such as agonistic stimulatory antibodies (CD137, OX40 or others). Using a similar approach as in TABs, negative checkpoint signals could be converted into immune activating signals, e.g., anti-CD47 × anti-CD19 (61), enabling phagocytosis and subsequent antigen presentation to T cells. Along the same lines, other bispecific antibody types such as the anti-angiopoetin-2 × anti-VEGF bispecific antibody RG7221, which can increase immune cell infiltration through vasculature normalization, might synergize with checkpoint blockade. An ongoing clinical study is currently investigating this question (NCT01688206), but no results on the combined activity have been reported yet (62). Current and upcoming clinical trials will provide data to expand the clinical value of such combination strategies and plans for further investigation and application.

## Author contributions

SP wrote the paper and prepared illustrations. FR wrote the paper. SK and JvB conceived the theme, wrote the paper, and prepared illustrations.

### Conflict of interest statement

The authors declare that the research was conducted in the absence of any commercial or financial relationships that could be construed as a potential conflict of interest.
